# Development of Risk Score for Predicting 3-Year Incidence of Type 2 Diabetes: Japan Epidemiology Collaboration on Occupational Health Study

**DOI:** 10.1371/journal.pone.0142779

**Published:** 2015-11-11

**Authors:** Akiko Nanri, Tohru Nakagawa, Keisuke Kuwahara, Shuichiro Yamamoto, Toru Honda, Hiroko Okazaki, Akihiko Uehara, Makoto Yamamoto, Toshiaki Miyamoto, Takeshi Kochi, Masafumi Eguchi, Taizo Murakami, Chii Shimizu, Makiko Shimizu, Kentaro Tomita, Satsue Nagahama, Teppei Imai, Akiko Nishihara, Naoko Sasaki, Ai Hori, Nobuaki Sakamoto, Chihiro Nishiura, Takafumi Totsuzaki, Noritada Kato, Kenji Fukasawa, Hu Huanhuan, Shamima Akter, Kayo Kurotani, Isamu Kabe, Tetsuya Mizoue, Tomofumi Sone, Seitaro Dohi, for the Japan Epidemiology Collaboration on Occupational Health Study Group

**Affiliations:** 1 Department of Epidemiology and Prevention, Center for Clinical Sciences, National Center for Global Health and Medicine, Tokyo, Japan; 2 Hitachi, Ltd., Ibaraki, Japan; 3 Teikyo University, Graduate School of Public Health, Tokyo, Japan; 4 Mitsui Chemicals, Inc., Tokyo, Japan; 5 YAMAHA CORPORATION, Shizuoka, Japan; 6 Nippon Steel & Sumitomo Metal Corporation Kimitsu Works, Chiba, Japan; 7 Furukawa Electric Co., Ltd., Tokyo, Japan; 8 Mizue Medical Clinic, Keihin Occupational Health Center, Kanagawa, Japan; 9 Mitsubishi Plastics, Inc., Tokyo, Japan; 10 All Japan Labour Welfare Foundation, Tokyo, Japan; 11 Azbil Corporation, Tokyo, Japan; 12 Mitsubishi Fuso Truck and Bus Corporation, Kanagawa, Japan; 13 Department of Safety and Health, Tokyo Gas Co., Ltd., Tokyo, Japan; 14 Health Design Inc., Tokyo, Japan; 15 Mizuho Health Insurance Society, Tokyo, Japan; 16 Fuji Electric Co., Ltd., Kanagawa, Japan; 17 ADVANTAGE Risk Management Co., Ltd., Tokyo, Japan; 18 National Institute of Public Health, Saitama, Japan; University of Louisville, UNITED STATES

## Abstract

**Objective:**

Risk models and scores have been developed to predict incidence of type 2 diabetes in Western populations, but their performance may differ when applied to non-Western populations. We developed and validated a risk score for predicting 3-year incidence of type 2 diabetes in a Japanese population.

**Methods:**

Participants were 37,416 men and women, aged 30 or older, who received periodic health checkup in 2008–2009 in eight companies. Diabetes was defined as fasting plasma glucose (FPG) ≥126 mg/dl, random plasma glucose ≥200 mg/dl, glycated hemoglobin (HbA1c) ≥6.5%, or receiving medical treatment for diabetes. Risk scores on non-invasive and invasive models including FPG and HbA1c were developed using logistic regression in a derivation cohort and validated in the remaining cohort.

**Results:**

The area under the curve (AUC) for the non-invasive model including age, sex, body mass index, waist circumference, hypertension, and smoking status was 0.717 (95% CI, 0.703–0.731). In the invasive model in which both FPG and HbA1c were added to the non-invasive model, AUC was increased to 0.893 (95% CI, 0.883–0.902). When the risk scores were applied to the validation cohort, AUCs (95% CI) for the non-invasive and invasive model were 0.734 (0.715–0.753) and 0.882 (0.868–0.895), respectively. Participants with a non-invasive score of ≥15 and invasive score of ≥19 were projected to have >20% and >50% risk, respectively, of developing type 2 diabetes within 3 years.

**Conclusions:**

The simple risk score of the non-invasive model might be useful for predicting incident type 2 diabetes, and its predictive performance may be markedly improved by incorporating FPG and HbA1c.

## Introduction

The prevalence of type 2 diabetes is rapidly increasing worldwide [1]. The International Diabetes Federation estimates that 382 million people had diabetes in 2013, and this number is expected to rise to 592 million by 2035 [1]. In addition, the number of those with prediabetes, a high risk state of developing diabetes, is projected to reach 472 million by 2030 [2]. Around 5–10% of individuals with prediabetes become diabetic every year, and up to 70% of those with prediabetes eventually develop diabetes [2]. In Japan, the prevalence of diabetes increased from 6.9 to 9.5 million between 1997 and 2012 [3]. Given that diabetes and associated complications decrease quality of life and represent a major health-care burden worldwide [2], detecting individuals at high risk of developing diabetes is important for prevention.

A number of risk factors for diabetes have been identified, including obesity, smoking habit, hypertension, level of physical activity, and family history of diabetes [4]. Numerous studies in Western populations have reported that weighted models and scores developed using these factors could identify individuals at high risk of developing diabetes [5]. Given the large differences in diabetes risk across ethnic groups [6], however, the performance of each model and score may differ by ethnicity [7]. In East Asia, several studies have developed risk models [8–15]. Of these, three studies were conducted in Japan [10, 11, 14] but did not include participants under the age of 40 [10, 11, 14] and did not validate the developed risk model [14]. Given the relatively high proportion of undiagnosed diabetes mellitus among younger age groups (under 40 years old) [16], models capable of identifying high-risk participants in this age group are still necessary. In addition, these scores require some variables including family history of diabetes and physical activity which are not routinely available or uniformly collected in general health examination in Japan, and thus are of limited use for wider population.

Here, we developed risk scores using only non-invasive risk factors initially and then incorporated laboratory measurements, such as fasting plasma glucose (FPG) and glycated hemoglobin (HbA1c), to predict 3-year incidence of type 2 diabetes. We then assessed the validity of the developed risk score in a large-scale multi-center study among Japanese workers.

## Methods

### Study Procedure

The Japan Epidemiology Collaboration on Occupational Health (J-ECOH) Study is an ongoing multi-center epidemiologic study among workers from several companies in Japan. A total of 12 companies covering various industries (electric machinery and apparatus manufacturing, steel, chemical, gas, non-ferrous metal manufacturing, automobile and instrument manufacturing, plastic product manufacturing, health care) participated in the J-ECOH study. In Japan, employees are obliged to undergo general health examination at least once a year under the health and safety law. As of August 2013, nine participating companies provided health check-up data obtained between January 2008 and December 2012 or between April 2008 and March 2013, which were combined to create an analytic database. The data of the earliest examination (mostly in 2008) was regarded as baseline, but if a 2008 dataset contained a large number of missing data, data from the 2009 examination was used as baseline (one company). We excluded data from one company due to a large number of missing data for both the 2008 and 2009 datasets.

### Ethics Statement

Prior to the collection of data, the conduct of the J-ECOH Study was announced in each company by using posters that explained the purpose and procedure of the study. Participants did not provide their verbal or written informed consent to join the study but were allowed to refuse their participation. This procedure conforms to the Japanese Ethical Guidelines for Epidemiological Research, where the procedure of obtaining consent may be simplified for observational studies using existing data. The study protocol including consent procedure was approved by the Ethics Committee of the National Center for Global Health and Medicine, Japan (NCGM-G-001140-05). Most participating companies provided data in either anonymized or de-identified form, but a few other companies provided data including identifiable information, which was removed from analytic database. The data are hosted in the National Center for Global Health and Medicine. Currently, the data cannot be widely shared because the research group has not obtained permission from participating companies to provide the data on request. However, the data can be requested by academic researchers for non-commercial research; inquiries and applications can be made to Department of Epidemiology and Prevention, Center for Clinical Sciences, National Center for Global Health and Medicine, Tokyo, Japan (Dr. Mizoue, mizoue@ri.ncgm.go.jp).

### Subjects

From a total of 82,380 participants in eight companies who received a health checkup in 2008 or 2009, 16,198 participants under 30 years old at baseline were excluded because the majority of them did not receive blood test, which is required for employees aged 35 years and 40 years or older by the Industrial Safety and Health Act in Japan. Of the remaining 66,182 participants aged 30 years or older, the present study included 53,216 participants who received health checkup 3 years after the baseline. Of these, we excluded participants who had diabetes at the baseline (n = 3512), who had missing information on glucose (n = 3192), HbA1c (n = 3274), or medical treatment of diabetes (n = 212), and who received blood sample in non-fasting status (n = 5321) or lacked information on fasting status (n = 1905). Some participants met more than one of the exclusion criteria. After further exclusion of 2031 participants with missing data for variables used to develop risk score of diabetes, including body height, body weight, waist circumference, blood pressure, smoking status, low-density lipoprotein (LDL) cholesterol, high-density lipoprotein (HDL) cholesterol, triglyceride, and drug use for hypertension or dyslipidemia, 37,416 participants (32,040 men and 5376 women) remained for analysis.

From the analytic cohort, we randomly selected a two-thirds derivation sample stratified by sex and site to develop a risk score for predicting incidence of diabetes (24,950 participants: 21,364 men and 3586 women). The remaining one third of participants (12,466 participants: 10,676 men and 1790 women) were included in a validation cohort to assess the validity of the derived risk score from the derivation cohort.

### Assessment of risk factors

Body height, body weight, and waist circumference were measured at each company in accordance with a standard protocol. Waist circumference was measured at the umbilical level in the standing position by a health professional. Body mass index (BMI) was calculated as weight in kilograms divided by squared height in meters. Smoking status and medication of hypertension or dyslipidemia were ascertained using a questionnaire. Blood pressure was measured using an automated sphygmomanometer. Hypertension was defined as systolic blood pressure of ≥140 mmHg, diastolic blood pressure of ≥90 mmHg, or taking medication for hypertension.

Biochemical measurements included plasma glucose, HbA1c, LDL-cholesterol, HDL-cholesterol, and triglycerides. Plasma glucose was measured by enzymatic method in seven companies and glucose oxidase peroxidative electrode method in one company. HbA1c was measured by latex agglutination immunoassay in five companies, high-performance liquid chromatography method in two, and enzymatic method in one. As HbA1c was measured in accordance with a method used by the Japan Diabetes Society, we converted values to the National Glycohemoglobin Standardization Program (NGSP) equivalent value (%) using the following formula: HbA1c (%) = 1.02 × HbA1c (Japan Diabetes Society) (%) + 0.25% [17]. In all participating companies, HDL-cholesterol, LDL-cholesterol, and triglyceride levels were measured by enzymatic method. Dyslipidemia was defined as LDL-cholesterol of ≥140 mg/dl, HDL-cholesterol of <40 mg/dl, triglyceride of ≥150 mg/dl, or taking medication for dyslipidemia. All laboratories involved in the health checkup in the participating companies have received satisfactory scores (rank A or score >95 out of 100) from external quality control agencies, including the Japan Medical Association, Japanese Association of Laboratory Medical Technologists, and National Federation of Industrial Health Organization.

### Outcome

Diabetes newly diagnosed during the 3-year period after the baseline examination was determined as the outcome in the present analysis. Diabetes was defined as having FPG of ≥126 mg/dl, or random plasma glucose of ≥200 mg/dl, HbA1c of ≥6.5%, or receiving medical treatment of diabetes, which was defined in two ways: medical treatment of diabetes (3 companies) or anti-diabetic drug use (5 companies). Individuals without diabetes at baseline who met any of these conditions in the subsequent health checkups were considered to have incident type 2 diabetes.

### Statistical analysis

The following variables were used to derive the risk score: sex, age (30–39, 40–49, 50–59, or ≥60 years), BMI (<21, 21-<23, 23<25, 25-<27, 27-<29, or ≥29 kg/m^2^), abdominal obesity (waist circumference ≥90 cm in men and ≥80 cm in women; yes or no), currently smoking (yes or no), hypertension (yes or no), dyslipidemia (yes or no), FPG level (<100, 100-<110, or ≥110 mg/dl), and HbA1c level (<5.6, 5.6-<6.0, or ≥6.0%). Data of characteristics in the derivation and validation cohort were expressed as means (standard deviation) and percentage for continuous variables and categorical variables, respectively. The differences in mean and percentage between derivation and validation cohort were tested using Student’s t-test and the chi-squared test.

Logistic regression analysis was performed to estimate OR and 95% CI of type 2 diabetes for each category of risk factors. We analyzed data using a multiple regression model with backward elimination methods with a significance level of less than 0.05 to first determine variables used for the non-invasive model (excluding dyslipidemia, FPG, and HbA1c). To develop a risk score for predicting 3-year incidence of diabetes, we assigned each category of risk factor with one of the following point scores, corresponding to β coefficients of multivariate logistic regression, in accordance with the method by Guasch-Ferré et al [18] and Lindström et al [19]: 1 for β = 0.01–0.20, 2 for β = 0.21–0.80, 3 for β = 0.81–1.20, 4 for β = 1.21–2.20, and 5 for β >2.20. The reference category of each variable was given a score of 0. The risk score of incident diabetes was calculated as the sum of the individual scores. We then assessed predictive performance for the risk score by drawing a receiver operating characteristic (ROC) curve, with calculation of the area under the ROC curve (AROC) and sensitivity, specificity, positive predictive value, and negative predictive value for various cutoffs. We also assigned point scores in accordance with methods in the Framingham [20] and Hisayama Study [10]. The score in the present study had a larger AROC than the score based on the Framingham Study’s method. While the score based on the Hisayama Study’s method showed better predictive performance than the present score in terms of AROC, the former had a much wider range than the latter. We therefore decided to use the present method to create a precise and simple risk score.

We next incorporated either or both FPG and HbA1c into the non-invasive model, thereby creating invasive models including FPG, HbA1c, or both. We performed ROC analyses for these models as well as the non-invasive model. We compared discriminative ability (AROC) among four models by using DeLong’s method [21]. In addition, we calculated net reclassification improvement (NRI) using three risk categories (<3%, 3–10%, and >10%) and integrated discrimination improvement (IDI). Finally, to assess the internal validity of the obtained risk scores, we applied the scoring system to the validation cohort and performed ROC analyses. We compared the predicted and observed incidence of diabetes in each decile of risk score and performed the Hosmer-Lemeshow test to assess model goodness-of-fit. Two-side *P* values of less than 0.05 were regarded as statistically significant. All analyses were performed using Stata version 13.0 (StataCorp, College Station, TX, USA) and Statistical Analysis System (SAS) software version 9.1 (SAS Institute, Cary, NC, USA).

## Results

Compared to participants included in the present analysis, those excluded were younger and more likely to be men and current smoker and to have hypertension and dyslipidemia, but had lower FPG and HbA1c for both age groups of 30–39 years (excluding 35 years) and ≥40 years (including 35 years) (data not shown).

During the 3-year period, we identified 1122 and 565 incident cases of diabetes in the derivation (1049 men and 73 women) and validation (537 women and 28 women) cohorts, respectively. The characteristics of study participants in the derivation and validation cohorts are shown in Table 1. The means (standard deviation) of age and BMI were 45.5 (7.9) years and 23.3 (3.2) kg/m^2^ in the derivation cohort and 45.5 (7.8) years and 23.3 (3.2) kg/m^2^ in the validation cohort. The means of age, BMI, waist circumference, and FPG and HbA1c levels and the proportion of women, current smokers, hypertension, and dyslipidemia did not differ markedly between the derivation and validation cohorts.

**Table 1:** Baseline characteristics of study subjects in the derivation and validation cohorts.

Characteristics	Derivation cohort	Validation cohort	*P* value^a^
No. of subjects	24,950	12,466	
Age (years)	45.5 ± 7.9	45.5 ± 7.8	0.85
Women (%)	14.4	14.4	0.97
BMI (kg/m^2^)	23.3 ± 3.2	23.3 ± 3.2	0.56
Waist circumference (cm)	82.2 ± 8.8	82.3 ± 8.8	0.85
Current smoker (%)	37.0	37.3	0.55
Hypertension (%)	16.6	16.4	0.57
Dyslipidemia (%)	45.1	44.7	0.52
FPG (mg/dl)	96.6 ± 9.1	96.5 ± 9.0	0.75
HbA1c (NGSP) (%)	5.53 ± 0.35	5.53 ± 0.35	0.87

Abbreviations: FPG, fasting plasma glucose; NGSP, National Glycohemoglobin Standardization Program. Data are mean ± standard deviation unless otherwise indicated.

^a^Based on chi-squared test for categorical variables and student t-test for continuous variables.

The association between risk factors and type 2 diabetes risk is shown in Table 2. In the non-invasive variables-adjusted model, men had significantly higher risk of type 2 diabetes than women. In addition, older age, higher BMI, abdominal obesity, current smoking, and hypertension were associated with an increased risk of type 2 diabetes. All variables remained significant in the backward elimination analysis (*P* value <0.05). In the model including dyslipidemia, FPG, and HbA1c, the ORs of type 2 diabetes for men, older age, higher BMI, abdominal obesity, and hypertension were considerably attenuated, such that sex, abdominal obesity, and dyslipidemia were no longer associated with type 2 diabetes risk. Participants with FPG of ≥110 mg/dl or HbA1c of ≥6.0% had significantly higher risk of type 2 diabetes than those with FPG of <100 mg/dl or HbA1c of <5.6%; the multivariable-adjusted ORs (95% CI) of type 2 diabetes were 13.69 (11.15–16.81) for FPG of ≥110 mg/dl or 17.26 (13.41–22.21) for HbA1c of ≥6.0%.

**Table 2:** Odds ratios and 95% confidence intervals of type 2 diabetes for each risk factor.

Risk factors	No of subjects	No of cases	Odds ratio (95% confidence interval)		
			Age-, sex-, and site-adjusted	Multivariable-adjusted (Non-invasive model)	Multivariable-adjusted (Full model)
**Sex**					
Women	3586	73	1.00 (reference)	1.00 (reference)	1.00 (reference)
Men	21,364	1049	2.31 (1.81–2.94)	1.85 (1.43–2.38)	1.13 (0.85–1.50)
**Age (year)**					
30-<40	6741	129	1.00 (reference)	1.00 (reference)	1.00 (reference)
40-<50	10,087	409	2.13 (1.74–2.61)	1.99 (1.62–2.44)	1.28 (1.01–1.61)
50-<60	7391	515	3.79 (3.11–4.62)	3.61 (2.94–4.43)	1.33 (1.06–1.68)
≥60	731	69	5.07 (3.74–6.88)	5.23 (3.81–7.17)	1.62 (1.12–2.32)
**BMI (kg/m** ^**2**^ **)**					
<21	5825	107	1.00 (reference)	1.00 (reference)	1.00 (reference)
21-<23	6589	195	1.38 (1.08–1.75)	1.36 (1.06–1.73)	0.99 (0.75–1.29)
23-<25	5963	274	2.08 (1.65–2.61)	1.97 (1.56–2.49)	1.15 (0.88–1.49)
25-<27	3670	260	3.29 (2.61–4.16)	2.81 (2.19–3.62)	1.36 (1.02–1.82)
27-<29	1674	131	3.95 (3.03–5.15)	2.99 (2.19–4.09)	1.39 (0.98–1.99)
≥29	1229	155	7.86 (6.06–10.19)	5.39 (3.90–7.45)	1.77 (1.22–2.58)
**Abdominal obesity** ^a^					
No	19,498	674	1.00 (reference)	1.00 (reference)	1.00 (reference)
Yes	5452	448	2.64 (2.33–2.99)	1.24 (1.03–1.50)	1.08 (0.88–1.34)
**Smoking status**					
Non smoker	15,714	636	1.00 (reference)	1.00 (reference)	1.00 (reference)
Current smoker	9236	486	1.24 (1.09–1.40)	1.30 (1.15–1.47)	1.27 (1.09–1.47)
**Hypertension**					
No	20,803	760	1.00 (reference)	1.00 (reference)	1.00 (reference)
Yes	4147	362	1.92 (1.67–2.20)	1.54 (1.34–1.77)	1.34 (1.14–1.58)
**Dyslipidemia**					
No	13,701	408	1.00 (reference)		1.00 (reference)
Yes	11,249	714	1.95 (1.72–2.21)		1.02 (0.88–1.19)
**FPG (mg/dl)**					
<100	16,489	156	1.00 (reference)		1.00 (reference)
100-<110	6252	322	5.28 (4.33–6.44)		2.96 (2.40–3.63)
≥110	2209	644	37.67 (31.13–45.57)		13.69 (11.15–16.81)
**HbA1c (NGSP) (%)**					
<5.6	12,731	91	1.00 (reference)		1.00 (reference)
5.6-<6.0	9725	326	4.96 (3.90–6.31)		2.90 (2.26–3.71)
≥6.0	2494	705	54.31 (42.86–68.82)		17.26 (13.41–22.21)

Abbreviations: FPG, fasting plasma glucose; NGSP, National Glycohemoglobin Standardization Program.

^a^Abdominal obesity was defined as waist circumference of 90 cm or more in men and 80 cm or more in women.

**Fig 1:**
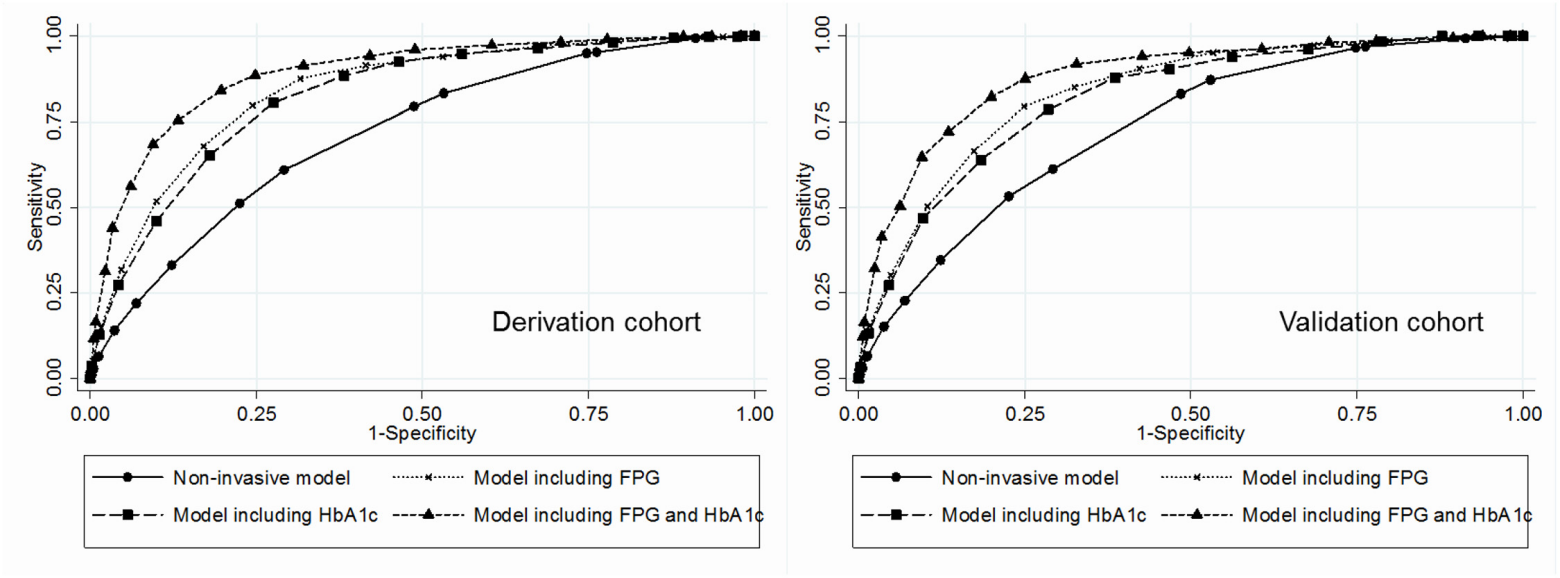
Receiver operating characteristics (ROC) curves for each risk score model in predicting type 2 diabetes. Abbreviation: FPG, fasting plasma glucose. In the delivation cohort, the area under the ROC (95% confidence interval) were 0.717 (0.703–0.731) for the non-invasive model, 0.843 (0.832–0.853) for the model including FPG, 0.827 (0.816–0.838) for the model including HbA1c, and 0.893 (0.883–0.902) for the model including both FPG and HbA1c. In the derivation cohort, the corresponding value were 0.734 (0.715–0.753) for the non-invasive model, 0.835 (0.820–0.851) for the model including FPG, 0.819 (0.803–0.835) for the model including HbA1c, and 0.882 (0.868–0.895) for the model including both FPG and HbA1c.

**Fig 2:**
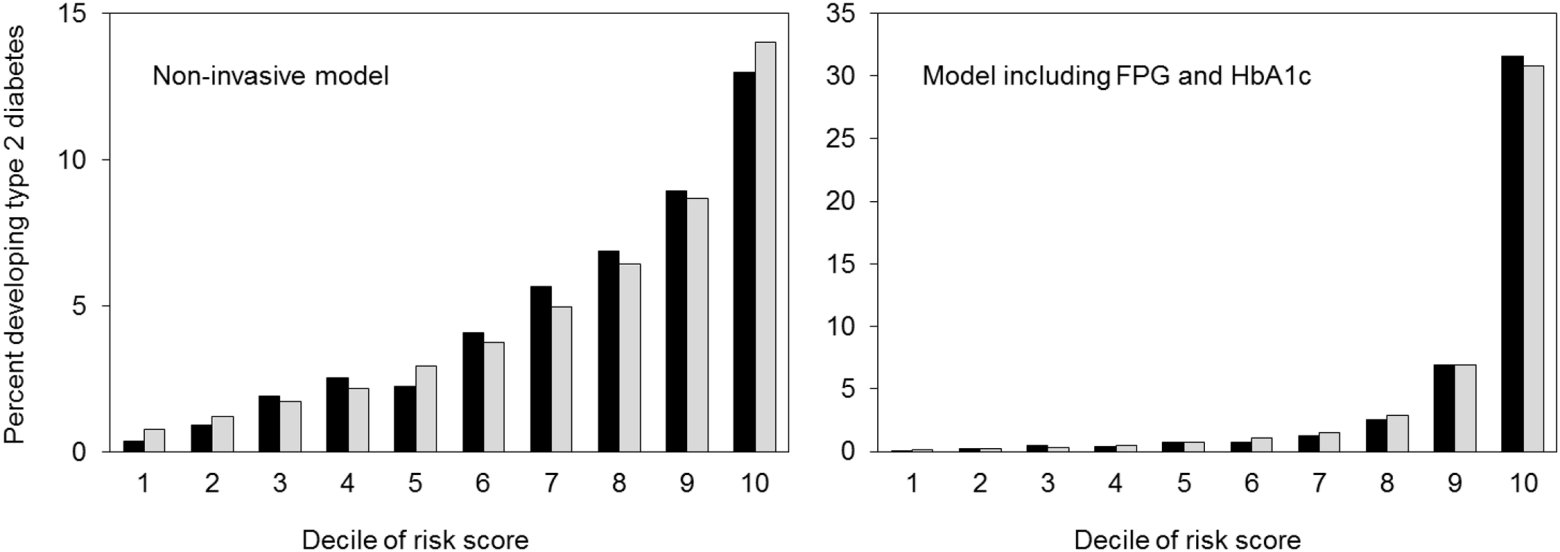
Observed and expected risk for developing type 2 diabetes (%) in each score of expected risk according to prediction models using the non-invasive model and the model including fasting plasma glucose and HbA1c in the derivation cohort; black bars, observed risk; grey bars, expected risk.

All risk factors in the four models and the points derived for each category are shown in Table 3. The total score ranged from 0 to 16 in the non-invasive model, from 0 to 15 in the invasive model including either FPG or including HbA1c, and from 0 to 20 in the model including both FPG and HbA1c. ROCs of each risk model in predicting type 2 diabetes are shown in Fig 1. The AROC for the non-invasive model was 0.717 (95% CI, 0.703–0.731), increasing to 0.893 (95% CI, 0.883–0.902) for the invasive model including both FPG and HbA1c (*P* value <0.001). In addition, the AROC for the invasive model including FPG (0.843) was significantly higher than that for the invasive model including HbA1c (0.827, *P* value <0.01) and was significantly lower than that for the invasive model including both FPG and HbA1c (0.893, *P* value <0.01). When AROCs were calculated by sex, they were larger in women than in men. The values (95% CI) were 0.746 (0.695–0.798) in women and 0.703 (0.688–0.718) in men for the non-invasive model and 0.898 (0.864–0.932) in women and 0.890 (0.880–0.900) in men for the model including both FPG and HbA1c. The NRI (95% CI) were 52.9% (49.5–56.4) for the invasive model including FPG, 53.2% (49.8–56.6) for the model including HbA1c, and 72.9% (69.7–76.2) for the model including both FPG and HbA1c as reference the non-invasive model. The IDI (95% CI) were 13.0% (12.3–13.6) for the model including FPG, 13.0% (12.4–13.7) for the model including HbA1c, and 21.0% (20.0–22.0) for the model including both FPG and HbA1c. The Hosmer-Lemeshow test showed a χ^2^ value = 19.3 and *P* value = 0.01 for the non-invasive model and χ^2^ value = 11.7 and *P* value = 0.17 for the model including both FPG and HbA1c (Fig 2).

**Table 3:** Points assigned to predict 3 year incidence of type 2 diabetes.

	Non-invasive model			Model including FPG			Model including HbA1c			Model including FPG and HbA1c		
Risk factors	β	OR (95% CI)	P	β	OR (95% CI)	P	β	OR (95% CI)	P	β	OR (95% CI)	P
**Sex**												
Women		1.00 (reference)	0		1.00 (reference)	0		1.00 (reference)	0		1.00 (reference)	0
Men	0.61	1.85 (1.43–2.38)	2	-0.10	0.91 (0.69–1.19)	0	0.70	2.01 (1.54–2.62)	2	0.12	1.13 (0.85–1.50)	1
**Age (year)**												
30-<40		1.00 (reference)	0		1.00 (reference)	0		1.00 (reference)	0		1.00 (reference)	0
40-<50	0.69	1.99 (1.62–2.44)	2	0.47	1.59 (1.28–1.98)	2	0.29	1.34 (1.08–1.66)	2	0.24	1.28 (1.01–1.61)	2
50-<60	1.28	3.61 (2.94–4.43)	4	0.69	2.00 (1.61–2.49)	2	0.49	1.63 (1.31–2.03)	2	0.29	1.33 (1.06–1.68)	2
≥60	1.65	5.23 (3.81–7.17)	4	1.11	3.03 (2.15–4.27)	3	0.49	1.64 (1.16–2.31)	2	0.48	1.61 (1.12–2.32)	2
**BMI (kg/m** ^**2**^ **)**												
<21		1.00 (reference)	0		1.00 (reference)	0		1.00 (reference)	0		1.00 (reference)	0
21-<23	0.30	1.36 (1.06–1.73)	2	0.08	1.09 (0.84–1.40)	1	0.12	1.13 (0.88–1.46)	1	-0.01	0.99 (0.76–1.29)	0
23-<25	0.68	1.97 (1.56–2.49)	2	0.36	1.44 (1.12–1.84)	2	0.34	1.40 (1.10–1.79)	2	0.14	1.15 (0.89–1.50)	1
25-<27	1.03	2.81 (2.19–3.62)	3	0.61	1.85 (1.41–2.42)	2	0.55	1.73 (1.32–2.26)	2	0.32	1.37 (1.03–1.83)	2
27-<29	1.10	2.99 (2.19–4.09)	3	0.67	1.96 (1.41–2.73)	2	0.47	1.60 (1.15–2.24)	2	0.34	1.41 (0.99–2.00)	2
≥29	1.68	5.39 (3.90–7.45)	4	1.16	3.20 (2.26–4.55)	3	0.73	2.08 (1.46–2.95)	2	0.58	1.79 (1.23–2.60)	2
**Abdominal obesity** ^a^												
No		1.00 (reference)	0		1.00 (reference)	0		1.00 (reference)	0		1.00 (reference)	0
Yes	0.22	1.24 (1.03–1.50)	2	0.16	1.18 (0.97–1.43)	1	0.09	1.10 (0.90–1.34)	1	0.08	1.09 (0.88–1.34)	1
**Smoking status**												
Non smoker		1.00 (reference)	0		1.00 (reference)	0		1.00 (reference)	0		1.00 (reference)	0
Current smoker	0.26	1.30 (1.15–1.47)	2	0.43	1.54 (1.34–1.77)	2	0.07	1.07 (0.93–1.23)	1	0.24	1.27 (1.10–1.47)	2
**Hypertension**												
No		1.00 (reference)	0		1.00 (reference)	0		1.00 (reference)	0		1.00 (reference)	0
Yes	0.43	1.54 (1.34–1.77)	2	0.19	1.21 (1.04–1.41)	1	0.50	1.64 (1.41–1.91)	2	0.30	1.34 (1.14–1.58)	2
**FPG (mg/dl)**												
<100					1.00 (reference)	0					1.00 (reference)	0
100-<110				1.54	4.66 (3.82–5.69)	4				1.08	2.96 (2.41–3.63)	3
≥110				3.49	32.85 (27.08–39.84)	5				2.62	13.70 (11.15–16.82)	5
**HbA1c (NGSP) (%)**												
<5.6								1.00 (reference)	0		1.00 (reference)	0
5.6-<6.0							1.52	4.55 (3.58–5.79)	4	1.07	2.90 (2.27–3.71)	3
≥6.0							3.81	45.05 (35.43–57.28)	5	2.85	17.33 (13.48–22.27)	5

Abbreviations: CI, confidence interval; FPG, fasting plasma glucose; NGSP, National Glycohemoglobin Standardization Program; OR, odds ratio; P, point.

^a^Abdominal obesity was defined as waist circumference of 90 cm or more in men and 80 cm or more in women.

The predictive performance for a range of cut-off points for the developed diabetes risk scores are shown in Table 4. In the non-invasive model, a score of 9 points or higher and where the sum of sensitivity and specificity was maximized in the derivation cohort had a sensitivity of 61.0% and specificity of 70.8%. In the model including FPG, a score of 7 points or higher with maximized sensitivity and specificity had sensitivity of 87.6% and specificity of 68.4%. In the model including HbA1c, a score of 10 points or higher had sensitivity of 80.7% and specificity of 72.4%. The model including both FPG and HbA1c with a score of 11 points or higher had sensitivity of 84.2% and specificity of 80.3%.

**Table 4:** Predictive performance of the developed diabetes risk scores.

	Derivation cohort							Validation cohort						
Risk scores	Sens	Spec	PPV	NPV	Ydn	LR+	LR-	Sens	Spec	PPV	NPV	Ydn	LR+	LR-
**Non-invasive model**														
≥5	95.3	23.7	5.6	99.1	19.0	1.25	0.20	97.0	23.6	5.7	99.4	20.6	1.27	0.13
≥6	95.0	25.2	5.6	98.3	20.2	1.27	0.20	96.6	25.1	5.7	98.7	21.8	1.29	0.13
≥7	83.3	46.7	6.9	98.2	30.1	1.56	0.36	87.3	47.0	7.2	98.5	34.3	1.65	0.27
≥8	79.5	51.2	7.1	97.5	30.7	1.63	0.40	83.2	51.4	7.5	97.5	34.6	1.71	0.33
≥9	61.0	70.8	9.0	97.1	31.8	2.09	0.55	61.2	70.8	9.0	97.2	32.0	2.09	0.55
≥10	51.3	77.5	9.7	96.5	28.7	2.27	0.63	53.3	77.4	10.0	96.6	30.7	2.36	0.60
≥11	33.0	87.7	11.3	96.2	20.9	2.70	0.76	34.7	87.6	11.7	96.2	22.3	2.80	0.75
**Model including FPG**														
≥5	94.0	46.9	7.7	99.3	40.9	1.77	0.13	95.2	46.6	7.8	99.2	41.8	1.78	0.10
≥6	91.4	58.5	9.4	99.2	50.0	2.20	0.15	90.4	57.8	9.2	99.0	48.2	2.14	0.17
≥7	87.6	68.4	11.5	98.8	56.0	2.77	0.18	85.1	67.5	11.1	98.7	52.6	2.62	0.22
≥8	79.8	75.5	13.3	98.2	55.3	3.26	0.27	79.5	75.1	13.1	98.1	54.5	3.19	0.27
≥9	67.9	82.9	15.8	97.5	50.9	3.98	0.39	66.6	82.7	15.4	97.4	49.2	3.84	0.40
≥10	51.8	90.0	19.6	96.7	41.8	5.19	0.54	50.3	89.7	18.7	96.6	39.9	4.86	0.55
≥11	31.8	95.3	24.3	96.1	27.2	6.83	0.72	30.3	95.2	22.9	96.1	25.4	6.24	0.73
**Model including HbA1c**														
≥5	98.2	21.3	5.5	99.5	19.5	1.25	0.08	98.4	21.3	5.6	99.4	19.7	1.25	0.07
≥6	96.5	32.6	6.3	99.5	29.1	1.43	0.11	96.1	32.3	6.3	99.4	28.4	1.42	0.12
≥7	94.9	44.0	7.4	99.4	39.0	1.70	0.12	94.0	43.8	7.4	99.2	37.8	1.67	0.14
≥8	92.6	53.5	8.6	99.1	46.1	1.99	0.14	90.4	53.1	8.4	99.1	43.6	1.93	0.18
≥9	88.4	61.7	9.8	98.8	50.2	2.31	0.19	88.0	61.4	9.8	98.6	49.3	2.27	0.20
≥10	80.7	72.4	12.1	98.0	53.0	2.92	0.27	78.6	71.5	11.6	98.0	50.0	2.75	0.30
≥11	65.2	82.0	14.6	97.3	47.3	3.63	0.42	64.1	81.5	14.1	97.3	45.6	3.47	0.44
≥12	45.9	90.1	17.9	96.6	36.0	4.63	0.60	46.9	90.3	18.6	96.5	37.2	4.81	0.59
**Model including FPG and HbA1c**														
≥5	98.3	29.2	6.1	99.7	27.5	1.39	0.06	98.2	29.2	6.2	99.6	27.4	1.39	0.06
≥6	97.4	39.5	7.1	99.6	37.0	1.61	0.07	96.3	39.3	7.0	99.6	35.6	1.59	0.09
≥7	96.1	51.1	8.5	99.5	47.2	1.96	0.08	95.2	50.3	8.3	99.5	45.5	1.91	0.10
≥8	94.3	57.9	9.5	99.4	52.2	2.24	0.10	94.2	57.3	9.5	99.4	51.5	2.21	0.10
≥9	91.4	67.9	11.8	99.3	59.3	2.85	0.13	91.9	67.2	11.7	99.2	59.1	2.80	0.12
≥10	88.7	75.1	14.4	99.1	63.8	3.57	0.15	87.6	74.9	14.2	99.0	62.5	3.50	0.17
≥11	84.2	80.3	16.7	98.7	64.5	4.27	0.20	82.3	80.0	16.4	98.5	62.3	4.12	0.22
≥12	75.6	86.7	21.2	98.4	62.3	5.70	0.28	72.2	86.5	20.2	98.2	58.7	5.33	0.32
≥13	68.5	90.5	25.4	97.9	59.0	7.24	0.35	64.8	90.5	24.4	97.6	55.3	6.80	0.39

Abbreviations: FPG, fasting plasma glucose; LR, Likelihood ratio; NPV, Negative predictive value; PPV, positive predictive value; Sens, sensitivity; Spec, specificity; Ydn, Youden index.

The 3-year predicted probability of incident type 2 diabetes by total points for each risk model are shown in Table 5. In the non-invasive model, participants with scores of 0 to 8 (69.4% of total participants in the derivation cohort) had less than 5% risk of 3-year incident type 2 diabetes. The risk were 5 to <10% for a score of 9 to 11 (23.0% of total participants), 10 to <20% for a score of 12 to 14 (7.0%), and >20% for a score of ≥15 (0.6%). In the invasive model including both FPG and HbA1c, participants with a score of 0 to 10 (77.4% of total participants), 11 to 12 (10.5%), 13 to 14 (6.9%), and 15 to 18 (5.0%) had less than 5%, 5 to <10%, 10 to <20%, and 35 to <50% risk of incident type 2 diabetes, respectively. Those with a score of ≥19 (0.2% of total participants) had >50% risk.

**Table 5:** Total point for each risk score and absolute estimated probability (%) of incidence of type 2 diabetes.

Non-invasive model		Model including FPG		Model including HbA1c		Model including FPG and HbA1c	
Score	Probability	Score	Probability	Score	Probability	Score	Probability
0	0.2	0	0.2	0	0.0	0	0.0
2	0.5	1	0.0	1	0.6	1	0.1
3	0.0	2	0.4	2	0.2	2	0.0
4	1.3	3	0.6	3	0.2	3	0.3
5	0.8	4	1.0	4	0.7	4	0.5
6	2.5	5	1.0	5	0.7	5	0.4
7	3.9	6	1.8	6	0.7	6	0.5
8	4.3	7	4.9	7	1.1	7	1.2
9	6.4	8	7.0	8	2.3	8	1.3
10	7.7	9	9.7	9	3.3	9	1.8
11	9.0	10	15.0	10	7.0	10	3.9
12	10.3	11	20.8	11	10.1	11	5.9
13	13.0	12	27.6	12	13.4	12	8.1
14	17.7	13	35.7	13	19.2	13	14.6
≥15	20.5	≥14	40.6	14	27.0	14	17.4
				15	44.2	15	35.1
						16	32.6
						17	45.1
						18	49.0
						19	53.3
						20	53.8

Abbreviation: FPG, fasting plasma glucose.

The ROCs of four risk scores in the validation cohort were closely similar to those in the derivation cohort (Fig 1). When AROCs were calculated by sex, values (95% CI) were 0.841 (0.756–0.927) in women and 0.708 (0.687–0.728) in men for the non-invasive model and 0.938 (0.896–0.981) in women and 0.872 (0.856–0.887) in men for the invasive model including both FPG and HbA1c. At the cut-off point for a non-invasive model score of 9 or higher, sensitivity (61.2%) and specificity (70.8%) were similar to those in the derivation cohort (Table 4).

## Discussion

In this large-scale, multi-center study among Japanese working population, we developed a risk score for predicting 3-year risk of type 2 diabetes. In the non-invasive model in which sex, age, BMI, abdominal obesity, smoking, and hypertension were included, the predictive ability for incidence of type 2 diabetes was reasonably good. Performance was further improved by adding FPG and HbA1c. Similar predictive ability was observed in the validation cohort. To our knowledge, this is the third largest study developing risk models and scores to predict incidence of type 2 diabetes.

The risk score based on the non-invasive model we developed showed relatively high predictive ability for type 2 diabetes. Noble et al [5] reviewed 94 risk models and scores and identified 40 non-invasive types of risk score, with AROCs ranging from 0.62 (for a model including BMI, waist circumference, and family history of diabetes) to 0.87 (for a model including age, BMI, waist circumference, use of antihypertensive drugs, history of hypertension, physical inactivity, and diet). The AROC for the non-invasive risk model in the present study was within this range (0.717; 95% CI, 0.703–0.731). Among Japanese studies, the Hisayama study established a non-invasive risk model using age, sex, family history of diabetes, waist circumference, BMI, hypertension, exercise habit, and smoking habit among 1935 participants aged 40–79 years [10]. In that study, AROC for the score in predicting incidence of type 2 diabetes (follow-up period, 14 years) was 0.700 (95% CI, 0.667–0.732). In the Toranomon Hospital Health Management Center Study 6 (TOPICS 6) among 7654 government employees aged 40–75 years [11], a non-invasive model including age, sex, family history of diabetes, BMI, and current smoking had an AROC of 0.708 (95% CI, 0.679–0.737) in predicting type 2 diabetes (follow-up period, 5 years). The risk score we developed in a large working population had comparable predictive ability but a more stable estimate (narrow confidence interval) than these previous Japanese studies. Given that 27% of the present study population were less than 40 years old, the present risk score could be applied to relatively young populations.

Numerous risk scores for diabetes have been developed across diverse populations. Given ethnic origin is strongly related to diabetes risk [2], however, a risk score derived from a population may not be applicable to others. In Japan, three diabetes risk scores have been developed: one among residents in a prefecture [14], another among residents in a rural town [10], and the other among government employees [11]. These scores require family history of diabetes, physical activity, and alcohol consumption which are not routinely available or uniformly collected in general health examination in Japan, and thus are of limited use for wider population. The risk scores we developed here using a large and multi-company database of periodic health checkup are not only statistically robust but also useful in practice.

We used six risk factors, including age, sex, BMI, abdominal obesity, smoking habit, and hypertension to create a non-invasive risk score. The number of factors used in risk models has ranged from 3 to 14 (mean 7.8) in previous studies [5]. The most commonly incorporated factors in previous risk models were age, family history of diabetes, BMI, hypertension, waist circumference, and sex, followed by ethnicity, fasting glucose level, smoking status, and physical activity [22]. We did not include family history of diabetes in the current risk model because that information was collected in only some participating companies. Nevertheless, the predictive power of our risk model was no less than those of previous risk models which did include family history of diabetes. To examine the impact of including family history of diabetes, we repeated the analyses in two major companies where this information was available (n = 31,202) and confirmed that AROCs for the non-invasive model were little improved after incorporating that information (from 0.724 to 0.736). Given that adults with family history of diabetes tend to have higher BMI, waist circumference, blood pressure, and FPG level than those without [23–26], a lack of family history information can be largely compensated by incorporating the above-mentioned data in the prediction model.

In the present study, the predictive power was enhanced by adding either FPG or HbA1c to the non-invasive model and was further improved by adding both, a finding compatible with previous studies [10, 11, 15, 18, 27, 28]. The degree of improvement was similar between the model including FPG and the model including HbA1c, though the difference was significant. In the TOPICS 6, the AROCs for the non-invasive models were increased to 0.836, 0.837, and 0.887 after FPG, HbA1c, and both FPG and HbA1c were added, respectively [11], and these corresponding values were 0.867, 0.886, and 0.893 in the European Prospective Investigation into Cancer and Nutrition-Potsdam study conducted in 1962 men and women aged 35–65 years [28]. The AROCs for the model including FPG were 0.772 in the Hisayama Study [10], 0.756 in a Thai cohort of 2677 participants aged 35–55 years [27], 0.784 in the PREDIMED study among 1381 participants aged 55–80 years [18], and 0.848 in a Taiwanese study among 36,972 participants aged 35–74 years [15]. The predictive ability of the present invasive model (AROC: 0.843 for FPG model, 0.827 for HbA1c model, and 0.893 for FPG and HbA1c model) was comparable to or higher than values in these previous studies. Further, the current model including both FPG and HbA1c can identify individuals at very high risk (≥40–50%) of developing type 2 diabetes within 3 years (Table 5) and thus is a tool for risk stratification.

Strength of the present study included a large number of participants from several companies, making our estimates highly stable (narrow confidence interval). In addition, we confirmed the performance of the risk score in a validation cohort. However, our study also has some limitations. First, we relied on HbA1c and fasting and casual glucose and not the oral glucose tolerance test to define incident type 2 diabetes. However, performing this test in a large sample is not feasible. HbA1c does not require fasting and reflects long-term glycemic status. In addition, the International Expert Committee has recommended using HbA1c to diagnosis diabetes [29]. Second, there were some differences in the characteristics between participants included and excluded from the present analysis. We thus could not rule out the possibility of selection bias as a result of these exclusions. Third, when we assessed the goodness-of-fit using the Hosmer-Lemeshow test, the *P* value for the non-invasive model was statistically significant, showing poor calibration. However, Hosmer-Lemeshow χ^2^ values <20 are considered indicative of good calibration [30] (χ^2^ = 19.3 and *P* value = 0.01 for the non-invasive model in the present study). Fourth, the follow-up period was 3 years, and thus the risk score developed may only be applicable to short-term prediction of diabetes. Nevertheless, the present score may serve for allocating resource to individuals who are at high risk of developing diabetes in near future. Fifth, the J-ECOH Study is a multi-company study using existing data, and thus survey questions regarding lifestyle, history of disease, and drug use as well as the procedure for obtaining anthropometric and biochemical measurement were not uniform. However, all laboratories that performed biochemical analyses for the participating companies have participated in one or more external quality control programs and received the highest rank of evaluation. Finally, most study subjects were workers in large companies; therefore, the present finding might not be applicable to workers in small- and medium-sized companies, those in other large companies with markedly different background, or members of the non-working population. Diabetes risk is probably higher in general population that includes vulnerable people than working population, and thus the application of the present score to the general population would lead to underestimation of diabetes risk. However, the extent of underestimation, if any, may be small, given a similar age-specific prevalence being reported in a nationally-representative sample [31].

In conclusion, we developed a simple risk model using age, sex, BMI, waist circumference, hypertension, and smoking status to predict 3-year incidence of type 2 diabetes in a large-scale multi-center study among Japanese workers. The predictive ability was reasonably high and well reproduced. Since this risk model uses non-invasive information, it may be useful among working population, particularly relatively young individuals who have less opportunity to undergo an examination of blood glucose levels than older individuals. Further, the risk model with FPG and HbA1c showed excellent predictive ability and thus can contribute to risk stratification, facilitating the development of efficient prevention programs against type 2 diabetes.
