# Correction: Development of Risk Score for Predicting 3-Year Incidence of Type 2 Diabetes: Japan Epidemiology Collaboration on Occupational Health Study

**DOI:** 10.1371/journal.pone.0199075

**Published:** 2018-06-07

**Authors:** Akiko Nanri, Tohru Nakagawa, Keisuke Kuwahara, Shuichiro Yamamoto, Toru Honda, Hiroko Okazaki, Akihiko Uehara, Makoto Yamamoto, Toshiaki Miyamoto, Takeshi Kochi, Masafumi Eguchi, Taizo Murakami, Chii Shimizu, Makiko Shimizu, Kentaro Tomita, Satsue Nagahama, Teppei Imai, Akiko Nishihara, Naoko Sasaki, Ai Hori, Nobuaki Sakamoto, Chihiro Nishiura, Takafumi Totsuzaki, Noritada Kato, Kenji Fukasawa, Huanhuan Hu, Shamima Akter, Kayo Kurotani, Isamu Kabe, Tetsuya Mizoue, Tomofumi Sone, Seitaro Dohi, for the Japan Epidemiology Collaboration on Occupational Health Study Group

There are errors present in the values presented in Table 5. Table 5 shows the observed probability. The corrected version of Table 5 now shows the estimated probability. The development and validation of the models is presented in Tables 2–4 and Figs 1–2 and these aspects of the work stand unaffected. The final "tool" that clinicians would be using is Table 5 to show the predicted probability that their patient with a given score will develop diabetes over the next 3 years. This is the culmination of the modeling work and has been reported incorrectly in the original publication. Please see the corrected Table 5 here.

**Table 5:** Total point for each risk score and absolute estimated probability (%) of incidence of type 2 diabetes.

Non-invasive model		Model including FPG		Model including HbA1c		Model including FPG and HbA1c	
Score	Probability	Score	Probability	Score	Probability	Score	Probability
0	0.5	0	0.2	0	0.1	0	0.1
2	0.9	1	0.2	1	0.1	1	0.1
3	1.1	2	0.4	2	0.2	2	0.1
4	1.5	3	0.6	3	0.2	3	0.2
5	1.9	4	1.0	4	0.4	4	0.3
6	2.5	5	1.6	5	0.6	5	0.5
7	3.2	6	2.5	6	1.0	6	0.7
8	4.2	7	3.9	7	1.6	7	1.1
9	5.4	8	6.1	8	2.4	8	1.7
10	7.0	9	9.4	9	3.8	9	2.5
11	9.0	10	14.2	10	6.0	10	3.9
12	11.5	11	21.0	11	9.2	11	5.8
13	14.5	12	29.8	12	13.9	12	8.6
14	18.2	13	40.4	13	20.4	13	12.6
15	22.6	14	52.0	14	28.9	14	18.1
16	27.7	15	63.4	15	39.3	15	25.4
						16	34.3
						17	44.4
						18	55.1
						19	65.3
						20	74.2

Abbreviation: FPG, fasting plasma glucose.

The twenty-sixth author’s name appears incorrectly. The correct name is: Huanhuan Hu.

There are several text errors throughout the article. The errors and corrections are as follows:

The last line of the Results paragraph of the Abstract section should read: Participants with a noninvasive score of ≥15 and invasive score of ≥ 18 were projected to have >20% and >50% risk, respectively, of developing type 2 diabetes within 3 years.

The last two lines of the sixth paragraph of the Results section should read: In the invasive model including both FPG and HbA1c, participants with a score of 0 to 10 (77.4% of total participants), 11 to 12 (10.5%), 13 to 14 (6.9%), and 15 to 17 (5.0%) had less than 5%, 5 to <10%, 10 to <20%, and 20 to <50% risk of incident type 2 diabetes, respectively. Those with a score of ≥18 (0.2% of total participants) had >50% risk.

There are errors in the figure caption for Fig 1. Please see the correct Fig 1 caption here.

**Fig 1:**
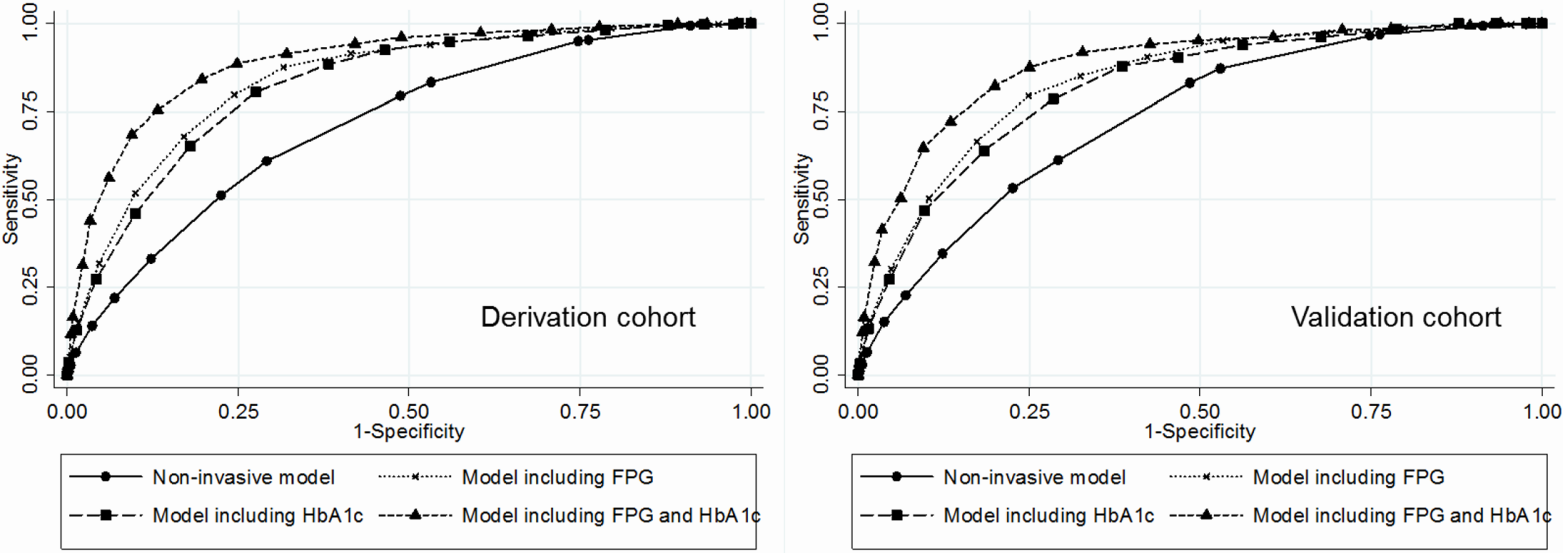
Receiver operating characteristics (ROC) curves for each risk score model in predicting type 2 diabetes. Abbreviation: FPG, fasting plasma glucose. In the derivation cohort, the area under the ROC (95% confidence interval) were 0.717 (0.703–0.731) for the non-invasive model, 0.843 (0.832–0.853) for the model including FPG, 0.827 (0.816–0.838) for the model including HbA1c, and 0.893 (0.883–0.902) for the model including both FPG and HbA1c. In the validation cohort, the corresponding value were 0.734 (0.715–0.753) for the non-invasive model, 0.835 (0.820–0.851) for the model including FPG, 0.819 (0.803–0.835) for the model including HbA1c, and 0.882 (0.868–0.895) for the model including both FPG and HbA1c.
